# General Practitioners and Chronic Non-Malignant Pain Management in Older Patients: A Qualitative Study

**DOI:** 10.3390/pharmacy4010015

**Published:** 2016-03-10

**Authors:** Mary-Claire Kennedy, Martin C. Henman, Gráinne Cousins

**Affiliations:** 1 School of Healthcare, University of Leeds, Leeds LS2 9JT, UK; 2 School of Pharmacy and Pharmaceutical Sciences, Trinity College Dublin, Dublin, Ireland; mhenman@tcd.ie; 3 School of Pharmacy, Royal College of Surgeons in Ireland, St. Stephens Green, Dublin , Ireland; gcousins@rcsi.ie

**Keywords:** chronic pain, general practitioners, elderly, primary care, analgesics, opioids

## Abstract

Chronic non-malignant pain (CNMP) is commonly managed by General Practitioners (GPs) in primary care. Analgesics are the mainstay of CNMP management in this setting. Selection of medications by GPs may be influenced by micro factors which are relevant to the practice setting, meso factors which relate to the local or regional environment or macro factors such as those arising from national or international influences. The aim of this study is to explore influences on GP practises in relation to pain management for older adults with CNMP. Semi-structured interviews were conducted with 12 GPs. Transcripts were organised using the Framework Method of Data Management while an applied thematic analysis was used to identify the themes emerging from the data. Clinical considerations such as the efficacy of analgesics, adverse effects and co-morbidities strongly influence prescribing decisions. The GPs interviewed identified the lack of guidance on this subject in Ireland and described the impact of organisational and structural barriers of the Irish healthcare system on the management of CNMP. Changes in practice behaviours coupled with health system reforms are required to improve the quality and consistency of pharmacotherapeutic management of CNMP in primary care.

## 1. Introduction

Chronic non-malignant pain (CNMP), has been defined as pain that persists for at least three months [1]. CNMP is a highly prevalent condition, estimated to affect 12%–30% of the European population with an increased prevalence observed in elderly populations [2,3,4]. This increased burden of CNMP among the elderly has been attributed to the development and progression of chronic degenerative conditions associated with the ageing process [5]. CNMP may be particularly debilitating for elderly patients, as, in addition to the physical and psychosocial features of the condition, it may also reduce mobility, produce gait disturbances, cognitive decline and result in accidents [5].

General practice is frequently the first and sometimes only point of contact with the healthcare service for patients with CNMP and analgesics are one of the primary management tools employed by General Practitioners (GPs) also known as primary care physicians [6,7]. The types of analgesics that may be prescribed for the patient may be influenced by the severity of the condition, the clinical appropriateness of the medication and the decision making process of the clinician [8,9]. Physiological alterations associated with the ageing process including multiple morbidities, cognitive decline and polypharmacy may limit the types of analgesics a clinician may use for pain management in older patients [9,10,11,12]. This can lead to an under-treatment of CNMP, a problem which is further exacerbated by the under-reporting of pain by older patients [13]. In the case of pain management, analgesic prescribing continues to be heavily guided by the WHO Analgesic Ladder despite being developed for the management of malignant pain. [14,15]. Opioids, which are included in Steps 2 and 3 of the ladder are valuable medications used in the management of CNMP. However, opioids may cause adverse outcomes in in elderly patient which may limit their use. There are also concerns surrounding their addiction potential and impact on health when used in the long-term [16]. These drawbacks are familiar to patients and GPs alike and may affect how these agents are used for the management of pain [17,18].

The range of factors that may influence a GP’s decision making process when prescribing medications have been classified as micro, meso and macro factors [19,20]. Micro factors include clinical considerations such as pain severity, co-prescriptions or comorbidities and concerns about potential adverse events in these patients which may result in suboptimal pain management [21,22]. The prescribing of NSAIDs and opioids presents particular clinical challenges for prescribers because of the adverse effects and cautions associated with these drug classes [21,22,23]. Other micro factors, such as the GP’s attitude towards CNMP, goals of pain management or concerns about professional scrutiny, may also produce variability in the prescribing of certain medications, e.g., opioids [8,24]. A GP’s confidence to prescribe analgesics may be influenced by their education and training in CNMP management during medical school, residency or through continuing professional development (CPD) [25].

Meso factors include guidance from local formularies, access to secondary or consultant care and cultural perceptions of the GP’s role. Barriers to effective pain assessment and management may arise from insufficient practice standards or difficulties in co-ordinating continuity of care within the healthcare system [23]. At present Irish patients may access care through the publicly funded system, through private payments, such as health insurance or out-of-pocket payments, or through a combination of public and private means. This system has been demonstrated to produce inequities in accessing healthcare, particularly services in high demand such as physiotherapy, occupational therapy (OT) or consultant led care [26]. The dynamic between the physician and patient may strongly influence the outcome of the consultation and is largely informed by cultural and societal perspectives of the role of physicians in the delivery of care. In westernised healthcare settings, such as Ireland, there is an increasing desire for a shared decision making approach in which consensus is reached between the patient and the GP in the development of an analgesic regimen [27].

Macro factors refer to the national and international context and are common to most GPs and include the availability of information and guidance on prescribing medications for CNMP [20]. The World Health Organisation (WHO) Analgesic Ladder (1986) provides a framework for analgesic prescribing although, as previously mentioned, the ladder has yet to be adapted for CNMP [15,28]. Clinical practice guidelines, conferences, journal articles or literature from pharmaceutical companies may also be information sources for GPs. Guidelines have been demonstrated to improve the quality of patient care and improve the cost-effectiveness of healthcare interventions when incorporated into a patient management plan [29]. However, there is evidence to suggest that clinical guidelines are not widely accessed by clinicians; barriers to their use include a lack of awareness and familiarity [30,31].

This study aims to explore influences on GP practices in relation to pain management for older adults with CNMP using the micro, meso and macro model as a framework to interpret the findings.

## 2. Methodology

### 2.1. Participants

This was a qualitative study of semi-structured interviews with GPs. GPs were recruited using a snowball sampling approach. Initially participants were identified through communication with the Health Research Board Centre for Primary Care Research, Royal College of Surgeons in Ireland. GPs were excluded from the study if they did not practice predominantly or exclusively in primary care or if they did not treat patients aged ≥65 years. The process of recruitment was finished when data saturation was reached *i.e.*, no additional themes were raised during the interview process [32].

### 2.2. Data Collection

All the interviews were conducted by MCK who is experienced in qualitative research. The interview guide detailing the questions posed to each participant is included in Table A1. Interviews were audio-recorded using the Audacity^®^ software and a portable recording device. GPs were supplied with a copy of the interview transcript and invited to edit the document. Following application of these revisions, an electronic copy of the transcript was transferred to NVivo^®^ Version 10. Ethical approval for this study was granted by the Health Research Ethics Committee, Trinity College Dublin.

### 2.3. Data Analysis

The Framework Method of Data Management facilitates the condensing of data through summarisation and synthesis of categories. The approach was developed in the 1980s by the National Centre for Social Research (UK) [33]. The NVivo^®^ Version 10 software package has incorporated a facility to arrange the data using the Framework Method. The Framework Method requires that data arising from the interviews is summarised and entered into a document resembling an Excel file; each row of the spreadsheet represents an interviewee, while each column is a theme or concept relevant to the transcript. Upon inputting a summary of the information or a direct quote from the transcript into the spreadsheet, it is possible to maintain a link to the original data source through electronically labelling and linking the data to the original transcripts that are uploaded on the programme.

Applied thematic analysis, used in this research, is an iterative approach which requires the researcher to reflect on the transcripts and consult with parties external to the analysis phase to ensure integrity of the process. [34]. Themes were developed by one researcher (MCK) and further reviewed and agreed by two researchers (MCK and MH). A theme is defined as “an abstract entity that brings meaning and identity to a recurrent experience and its variant manifestations” [35]. Themes may be subcategorised as follows: “basic”, this lowest order of understanding emerging from text, “organizing” which are categories of basic themes combined which produce an abstract principle or “global” which represent principle metaphors of the dataset. Upon completion of this phase of analysis, a second member of the research team (MH) reviewed a sample of the transcripts to ensure that coding and thematic analysis was complete and exhaustive. The reflexivity of data analysis was enhanced by extensive documentation and auditing of the data management phase and analytical processes and also by allowing a second researcher to review the data analysis [36,37].

## 3. Results and Discussion

### 3.1. Results

Twelve GPs were interviewed, four interviews were conducted in person and eight by telephone. The average duration of interviews was 25 min. Participating GPs practised in both rural and urban settings and had been working in general practice for between 3 and 30 years. All GPs completed their undergraduate education in an Irish university.

#### 3.1.1. Micro Factors

The aetiology, severity and progression of pain and the potential risks or benefits associated with analgesic and adjuvant medications were identified as the key micro factors influencing GP prescribing. The risk-benefit analysis described by GPs involved comparing the efficacy of medications with potential adverse outcomes. Cautions and contraindications arising from the patient’s co-morbidities or medical history contribute to the assessment upon initial and subsequent consultations with older patients. When asked if a validated pain scale was used during patient consultations, all GPs replied that they did not incorporate scales into their everyday practice. Rather, the majority of GPs described establishing pain severity based on impact on daily activities:

I don’t use standardised scales for pain, it is often volunteered by the patient who comes and says they are in pain and then over time it gets to where a pattern is established where certain medication is being prescribed regularly.

(GP9)

The process of consulting with the patient to establish the nature and severity of pain may be considered a cyclical process, as the GP considers these variables with each consultation or prior to initiating a new analgesic. GPs described regular review of the patient’s medication to determine on-going efficacy and suitability of the medication:

I always review them…I don’t necessarily need them to come to me, they can sometimes call me by telephone and tell me how they’re getting on…

(GP12)

GPs discussed the appropriateness of a medication in terms of a risk-benefit assessment for that patient. The patient’s medical history, psychosocial background, co-morbidities, co-prescriptions and adverse outcomes were central to this decision making process, balanced with the pain relief attained. It became apparent that the process of prescribing analgesics was quite challenging given the significant potential for adverse outcomes with older patients in particular.

It’s a very fine balancing act, I mean you have to take into consideration that there are serious side effects, but on the other hand sometimes if their pain isn’t well controlled then you have no choice but…to step up the ladder and put them on stronger painkillers.

(GP12)

Analgesic efficacy were discussed in the context of the WHO Ladder; if an adequate response was not achieved by a medication, the next step in the ladder was considered. However, GPs described their efforts to maintain the patient on Step 1 of the ladder or attempts to de-prescribe stronger analgesics once pain relief was attained or acute exacerbations were overcome:

Usually I would start with an NSAID if it’s OA [osteoarthritis] type pain or I usually do try to get people onto regular paracetamol 1gr tds [three times daily] ongoing but I find that people don’t have much faith in it…and they have usually tried it and even if it’s in conjunction with something else, they will usually be not keen on starting it [pause] but ideally I would start them on paracetamol and move up to an NSAID and then onto something like tramadol.

(GP2)

GPs described a conservative approach to prescribing opioids for older patients and listed concerns including clinical appropriateness and the risk of side-effects including sedation, gastrointestinal (GIT) effects, tolerance and addiction as the reasons for reluctance in initiating these agents:

If I am needing to use something in the longer term then I would try to use a combination low dose, a paracetamol with a codeine. So I would probably step up to something like Solpadeine^®^ [paracetamol 500 mg/codeine 8 mg/caffeine 30 mg] to see if that would help and try to get them to come back and review in two weeks to see where they are at.

(GP4)

I tend to be careful with codeine based medicines really. I don’t tend to use codeine a lot particularly in the elderly because it is nauseating and constipating and sedating and a lot of other things.

(GP7)

If they’re not getting any relief from paracetamol on its own, usually I would step to non-steroidal but you have to be really careful with elderly people and non-steroidals …then we’d go on and move onto the Tylex^®^ [paracetamol 500 mg/codeine 30 mg] and the codeine based analgesics. Unfortunately, they’re very poorly tolerated by elderly people.

(GP12)

There were a number of different practices described by GPs for the co-prescribing of laxatives with opioids. All GPs were aware that it is good practice to concurrently prescribe laxatives with opioids, particularly moderate and stronger opioids, but a number admitted that this was occasionally overlooked in practice:

I should and I know I should but yet I don’t always.

(GP9)

I am probably not as good with the Tylex^®^ [paracetamol 500 mg/codeine 30 mg] though I should always prescribe lactulose but if I am moving somebody onto a patch at all they will always get a co-prescription for a laxative and if I remember it at all they will get a co-prescription when they are going on Tylex^®^ but probably not as good at the co-prescribing for the laxative with the Tylex^®^ to be honest.

(GP4)

GPs repeatedly described the necessity to review opioid prescriptions so as to minimise long-term use and adverse outcomes, viewing the process as a battle rather than a collaboration with the patient:

Battling to try and keep people on the lower end of the scale so you know encourage them to stay with regular paracetamol qds [four times daily], or moving to Solpadeine^®^ [paracetamol 500 mg/codeine 8 mg/caffeine 30 mg], so I would rarely move to something higher than that until you get into I guess what you are talking about, chronic pain patients.

(GP9)

If pain relief could not be attained by Step 2 analgesics, GPs described cautiously moving to Step 3 opioids. However, a number of GPs indicated that they would refer the patient to a consultant at this point:

For non-malignant chronic pain I would move up to tramadol and then I would add in probably at that point then amitriptyline or pregabalin. I generally [pause] I wouldn’t go much beyond [pause] in terms of me initiating the treatment I wouldn’t go much beyond tramadol.

(GP6)

I would be quite cautious about starting patients on those, particularly for what appears to be a (pause) what is likely to be a chronic problem. I do prescribe them but with a lot of reservations particularly Solpadol^®^ [paracetamol 500 mg/codeine 30 mg] and Ixprim^®^ [tramadol 37.5 mg/paracetamol 325 mg] things like that.

(GP6)

Patches were the preferred method of administration of strong opioids. GPs described being comfortable maintaining the patient on these formulations, with continuous review:

I did find the arrival of the opiate patches a huge relief to my stress over managing people who were going to have ongoing, continuous pain and we really have to put something significant in place on an ongoing basis.

(GP11)

If I’ve put somebody on a BuTrans^®^ [buprenorphine] patch, a low dose once a week and their pain is well controlled on that, I would leave them on that indefinitely.

(GP12)

A history of substance addiction or misuse is a caution for prescribing opioids in patients presenting with pain; this was stated by a few GPs as a key consideration when initiating these agents:

You would want to be sure it was going to be short term…so you would want to know there is no history of any addiction.

(GP5)

The potential for adverse effects and cautions for use associated with NSAIDs were also discussed by GPs. All GPs described their reservations in using systemic NSAIDs in older patients. Those that incorporated NSAIDs into the analgesic regimen were content to do so only for short periods:

I try to avoid NSAIDs in the elderly except for very short periods of time obviously because of the risk of renal impairment and GI stuff but I will use them for short periods of time in people who don’t have contraindications.

(GP4)

The perceived risks associated with NSAIDs were contrasted with those of opioids particularly those containing higher doses of codeine and tramadol:

Ideally we are trying to avoid any of the kind of dependency drugs, any of the opiates whether they be low or high dose and then even now trying to avoid anti-inflammatories because of the cardiovascular side effects.

(GP9)

GPs reported incorporating adjuvants into an analgesic regimen if they identified neuropathic pain but were selective about the type of agent to use in older patients:

I identify with amitriptyline being a potentially inappropriate drug in an older population [pause] amitriptyline would be bad…probably go with, for neuropathic pain, pregabalin or gabapentin.

(GP9)

#### 3.1.2. Meso Factors

There was a consistent sense of the fragmented nature of the Irish healthcare system as GPs discussed their experiences with referral of patients to other healthcare professionals and the difficulties associated with accessing services, particularly for patients receiving public care. These concerns were particularly highlighted by GPs in rural areas. The opportunity to refer a patient to a multidisciplinary team with specialised knowledge in primary care would be a welcome development for GPs, who currently almost exclusively oversee patient care:

It’s kind of silo medicine, but that’s an awful lot of Irish medicine is silo medicine, and I think a lot of the primary care teams are only teams in name.

(GP10)

We don’t have primary care teams on the ground that are supposed to be…I wish there was a team we could refer them to that would look after them, but there isn’t. You can do a Physio referral or you can get the PHN [public health nurse] to call, it’s very disjointed.

(GP12)

A number of GPs described seeking assistance from consultants and pain clinics for patients when adequate analgesia was not achieved, or when a diagnosis was difficult to achieve in primary care. While the majority of GPs were satisfied with the quality of care received from specialists, access to these services for public patients was described as being difficult by the majority of GPs. Geographical proximity to a pain clinic was also cited as a limiting factor in gaining access to specialised interventions.

There is a dedicated pain clinic…the waiting lists though are hugely variable. Obviously if somebody has private insurance they can be seen quicker, but lots of elderly people don’t and the waiting lists can be quite prohibitive.

(GP4)

(Access is) very bad unless you have a thing called money and you could easily be waiting a long long time if you have a medical card to see a pain specialist, it is a very under-staffed under resourced [pause] unless you have money...A very bad system and a very unfair system unless you can afford it.

(GP5)

So often if you can get the pain clinic on board as well they tend to have a good multidisciplinary approach. The pain nurse can often be quite good [pause] just other alternative regimes can often work.

(GP4)

Several references were made to initiation of Step 2 analgesics in Accident and Emergency (A & E) or for post-surgical patients. A few GPs expressed surprise at the types of analgesics prescribed for these patients; the appropriateness of such regimens; and the challenges presented in discontinuing the regimen:

I’m not a big fan of Tylex^®^ [paracetamol 500 mg/codeine 30 mg] and I’m just bemused at the volume and dosage that comes out of casualty in particular...

(GP11)

GPs were asked to comment on the extent to which physiotherapy or additional interventions, such as OT, were accessed in the community setting and their experiences with these services. GPs appeared to place these services at the centre of their patient management plan however access to physiotherapy was problematic due to the length of public waiting lists and cost for private patients. All GPs expressed the view that limited access to such services in primary care placed the burden on them to manage patients with analgesics whilst awaiting these services:

If we had access to community occupational therapy and community physiotherapy I think we would prescribe way less, I strongly believe that. The big big issue is not having anything to offer anybody and having these huge waits for everything I definitely think if we had a more multidisciplinary input prescribing, would be way down.

(GP4)

#### 3.1.3. Macro Factors

All GPs indicated that their understanding of the pharmacotherapeutic management of older patients with CNMP was adequate to effectively manage patients and that they would be capable of seeking out additional information on the subject if required. There was no difference noted between awareness or use of clinical guidance and the number of years the GP was in practice with both recently qualified GPs and those practicing for many years incorporated some form of guidance into the prescribing decision making process. The absence of specific literature or guidelines on prescribing for CNMP in the Irish primary care setting was acknowledged by a number of GPs. A number stated that guidelines would be useful in practice but highlighted that management strategies may vary between GPs according to a patient’s access to the healthcare system and the consultant care provided therein. While guidelines might be a welcome resource for GPs, it was highlighted that guidance needs to be succinct and focused so as to be of practical use.

It is probably an area to the best of my knowledge we don’t have much in the way of guidelines for non-malignant elderly pain guidelines that have come across my desk anyway.

(GP7)

Definitely a booklet on non-malignant pain would be fantastic but with the caveat that with the non-pharmacological stuff, your access will depend on where you work.

(G4)

There are two problems, we are overloaded with information and paper and everything else in practice and then we want clarity.

(GP3)

CPD events such as lectures and workshops provide GPs with the opportunity to enhance their knowledge on a range of topics:

I was at one…which was run by the Pain Society of Ireland and it was over two days and they had a number of speakers talking about managing pain in primary care.

(GP4)

The majority of GPs reported accessing clinical practice guidelines on the topic of palliative pain management and adapting them for CNMP management:

There is also palliative care produced guidelines [pause] I know it’s for cancer but they tend to be very good at producing guidelines if I am prescribing morphine in terms of what your equivalent dosing is for different types of morphines. So I would tend to use palliative guidelines if I am actually prescribing morphine just in terms of dosing and stepping up and stepping down.

(GP4)

The WHO Ladder was referred to by all GPs as the basis for prescribing analgesics:

So the one that everyone refers to is the WHO Analgesic Ladder, so ideally we are trying to avoid any of the kind of dependency drugs.

(GP9)

I would use the WHO pain ladder; so starting off with a simple analgesic then moving up to NSAIDs.

(GP6)

The British National Formulary (BNF) was cited as the primary guidance for all GPs in day-to-day practice, while three GPs listed peer-reviewed journals and educational websites as resources for updates on recent clinical developments:

Another good website is The American Academy for Family Practitioners; they have tonnes of information on their website and another lot in California called Audiodigest. I download a lot and listen to it on my IPhone during my rounds.

(GP5)

I would look up, there’s various kind of websites, the NHS [National Health Service] have a very good one, it used to be called PRODIGY Clinical Knowledge Summaries.

(GP10)

Literature provided by pharmaceutical companies was mentioned by a number of GPs. These GPs spoke negatively about the focus and tone of this information, stating that it may be misinterpreted due to the way the data is presented:

I don’t think you are supported at all except by the drug companies who are mad keen to support you.

(GP8)

I have reservations about pregabalin and Lyrica^®^ [gabapentin] because it seems in a way [pause] it is just being pushed by pharmaceutical companies as super effective whereas, in reality, it isn’t any more effective.

(GP9)

### 3.2. Discussion

This study aimed to explore influences on GP practices when prescribing analgesics for older adults with CNMP. The interviews with these GPs reinforced existing knowledge from the literature on this subject, such as the higher prevalence of CNMP among older people and the significant psychosocial and economic burden of the condition [9]. This study deepens our understanding of this subject and also enhances our knowledge of how micro, meso and macro factors relate to the prescribing of analgesics for older patients with CNMP. These factors have not previously been shown to apply to an Irish context.

Micro factors include patient-specific clinical considerations such as pain severity, medical history, comorbidities and co-prescriptions. Analgesic prescribing was viewed as a compromise between minimising adverse outcomes and providing pain relief. These restrictions may lead to under-treatment of pain among older patients, a problem that the GPs acknowledged but appeared willing to accept, following a risk-benefit assessment. Meso and macro factors, such as the availability of prescribing information and access to additional interventions in primary and tertiary care also informed management by the GP. However, these factors appeared to influence prescribing to a lesser extent than micro factors. Thus, although the Irish healthcare system differs substantially in its structures and operation from other European countries, it was the micro factors relating to the patient that appeared to most influence GPs.

The GPs in this study advocated a conservative approach to analgesic prescribing, attempting to remain on the lowest step of the analgesic ladder for the longest possible duration. Paracetamol and NSAIDs were preferred first line analgesics, prescribed when possible and appropriate. However, GPs described a poor acceptance of paracetamol as an effective analgesic by patients. This lack of acceptance of paracetamol as an analgesic has been identified in several studies and presents a significant problem to clinicians when devising an analgesic regimen for patients [38,39]. The appropriateness of oral NSAIDs for elderly patients was also discussed by GPs in this study who expressed concern about the safety of this drug class. These concerns are widely referenced by GPs in the literature and further restrict the types of analgesics that may be considered for this group of patients [40,41,42].

“Opiophobia” describes reluctance among physicians to prescribe strong opioids [43,44]. This has been attributed to poor knowledge and experience of opioids, lack of education in pain management, fear of regulatory scrutiny or concerns about adverse outcomes [45,46]. “Opiophobia” in the Irish context appears to be associated with concerns relating to adverse effects of opioids and not from professional or regulatory scrutiny, which has been cited as a concern of North American physicians [47]. The negative consequences, such as sedation, cognitive impairment and GIT effects, were extensively discussed by GPs as a barrier to the initiation of opioids and were reserved for those patients with severe pain or when a diagnosis has been established. This is a similar finding to a survey of UK GPs (*n* = 115) in which opioids were viewed as a last resort for CNMP management [48]. However, a subset of GPs in this study identified transdermal agents as an acceptable route of administration of strong opioids to older patients. This differs from a Canadian study which indicated that GPs were less comfortable with prescribing fentanyl patches in comparison to oral opioids [49]. While a number of GPs described concerns about opioid addiction and misuse, several stated that these were not considerations with older patients. This differs to the findings of a number of qualitative and quantitative studies of other patient groups which have demonstrated that these considerations influence prescribing [25,48,50,51,52]. However, one study which focused on older adults with CNMP reported findings similar to those described here [45].

The lack of national prescribing guidance for CNMP was acknowledged by the GPs. Several described accessing such information on websites or in evidence-based journals while others used literature published by pharmaceutical companies. However, several GPs spoke skeptically of the manner in which evidence was presented in literature from pharmaceutical companies. While the prospect of prescribing guidelines was welcomed by several GPs, the problems associated with implementation of guideline recommendations in primary care were highlighted. This aligns with the literature which suggests that lengthy guidelines which are poorly adapted for the practice setting impair implementation [30,53]. Multimodal management is not required for all patients with CNMP, but those patients that require such interventions may benefit from physiotherapy, OT or psychological based therapies, as acknowledged by GPs in this study ([54], pp 24–29). Access to physiotherapy and OT in primary care, and pain consultants or pain management programmes in tertiary care, are associated with better outcomes and quality of life and are widely viewed as essential in a pain management plan [55,56]. There was an apparent frustration with access to these services and a sense that the disjointed nature of the Irish system places pressure on GPs to act as gatekeepers in the provision of care to CNMP patients. The limited access to additional interventions, further restricts a GPs ability to provide beneficial interventions to supplement analgesic therapies [57,58].

There are a number of strengths and limitations to this research study. The method of recruitment has the potential to introduce selection bias, as individuals volunteered to contribute to the research. Interviewing fellow healthcare professionals can be advantageous as technical terms are easily communicated and it removes the likelihood of misinterpretation. However, this may also have introduced bias into the study if GPs felt unable to differ from professionally acceptable responses, perhaps not disclosing unacceptable practices. A number of the interviews were conducted by telephone which causes the loss of nonverbal cues gained from face-to-face interaction and the inability to use visible aids during the discussion. However, telephone interviews may ease the interaction between individuals particularly when discussing subjects that may be sensitive for the interview [59]. In addition, telephone interviews are inexpensive and time-efficient for the researcher and participants [60,61].

The findings of this research have potential implications for practice at the micro, meso and macro levels as they relate to primary care and for pain research. The reported absence of a formal pain assessment approach may lead to a lack of understanding by the GP of the severity of the patient’s pain, which may contribute towards inadequately managed pain [23]. While it is not possible to objectively assess pain, the introduction of a standardised assessment tool may aid GPs evaluate patients in a systematic manner. The issue of “opiophobia” may best be addressed through further education of GPs and introduction of a clinical guideline for GPs to support decision making processes when prescribing opioids [62]. Educational interventions may enhance knowledge of pain management strategies, allay concerns about adverse outcomes arising from pharmacotherapy and help GPs explore the balance between the risk and benefits of drug treatment with older patients. Such interventions may influence micro and meso level factors but further research of the effectiveness of such interventions is required with small GP groups before implementation on a larger scale.

The current reliance on GPs in primary care to independently manage or navigate the patient through the healthcare system to a pain specialist is a point of concern for clinicians. This feature of the healthcare system which influences prescribing at a macro level may be addressed through the provision of enhanced services in primary care to support GPs. A multidisciplinary primary care team, consisting of GPs, physiotherapists, OTs and public health nurses, was described by many GPs as a possible solution to the current challenges in accessing specialised care. Such a team in primary care would allow patients to gain more rapid access to a group of physicians and allied healthcare professionals for day-to-day management and has been demonstrated to improve clinical outcomes for patients with CNMP [63].

## 4. Conclusions

This study provides an insight into the factors influencing the prescribing of analgesics for older patients with CNMP in primary care. The selection of an analgesic regimen is dependent on a broad range of micro, meso and macro factors relating to the prescribing physician, the patient, and influences from external sources, such as the availability of guidelines and evidence-based literature. Clinical considerations including patient history, comorbidities, co-prescriptions and pain severity are key factors informing the development of an analgesic regimen. The remaining factors such as access to prescribing information, interaction with the patient and health system also inform the approach to management adopted by the GP. However, these factors appear to be weaker motivators in the prescribing process than the clinical considerations but nonetheless appear to influence the pharmacotherapeutic management of the patient in primary care.

## Appendix A

**Table A1:** Interview Guide.

Question Theme	Statements/Questions in Interview Guide
Experiences and Attitudes in the Prescribing of Analgesics	Do you treat many patients aged 65 and older for chronic pain conditions in your practice? What are the most common types of pain conditions you see on a day-to-day basis? What is your approach for managing patients aged 65 and older with chronic non-malignant pain conditions in your practice? How does this contrast with your management of pain in younger populations?Assessment? Initiation on painkillers? Do you feel confident managing these patients in the primary care setting? (*Prompt: access to specialist management*) Do you feel confident prescribing analgesics for these patients? At what point in the management of a patient’s pain condition do you consider initiation of an opioid analgesic medication? How does this contrast with your management approach in you younger populations?
Support Available to GPs	What is your opinion of the support available to GPs in the management of CNMP patients, *i.e.*, referral to physiotherapists, occupational therapists, pain clinics, *etc.*? *(Prompts: Ease, Cost-Effectiveness)*
Prescribing Analgesics for Elderly Populations	Do you prescribe opioid type medications for patients with chronic pain conditions? Can you tell me about the circumstances under which you are more likely to prescribe an opioid analgesic? Some patients wish to continue taking opioids on a long-term basis. What are your thoughts on prescribing opioid analgesics on a continuous basis for your patients? At what point do you decide to change from one opioid formulation to another? *(Prompt: Is this a consistent approach for all patients?)* Are there any circumstances under which you would not prescribe an opioid analgesic to a patient? What is your approach to discontinuing a patient from an opioid analgesic?
Patient-GP Interactions	Do you think that your patient’s expectations of a doctor’s visit influence your prescribing of analgesics?
Adverse Effects Associated with Opioids and Management of Adverse Effects	Are you concerned about the adverse effects such as constipation and nausea arising from opioid use? Do you take steps to prevent/manage such adverse effects? For instance do you prescribe laxatives when you prescribe opioids? Do you consider the sedative medications such as sleeping agents and benzodiazepines that the patient might be taking when you prescribe opioid medications?
Clinical Practice Guidelines and Prescribing Information	*In some countries such as the United States and Canada there are many clinical practice guidelines available. These clinical practice guidelines provide advice to physicians on the types of analgesics that are appropriate for the management of different types of pain conditions and the steps that should be taken to ensure optimal management whilst minimising risks associated with therapy* Do you consult guidance on prescribing such as the BNF or other guidance before selecting an opioid formulation for a patient? If so what guidance? Do you think that such guidance would be useful to you in your everyday practice? Who do you think should issue this guidance?

## References

[1] Carr D., Fricton J., Jadad A., Lipkowski A., McGrath P. (2003). How prevalent is chronic pain?. Pain Clin. Updates Int. Assoc. Study Pain.

[2] Breivik H., Collett B., Ventafridda V., Cohen R., Gallacher D. (2006). Survey of chronic pain in europe: Prevalence, impact on daily life, and treatment. Eur. J. Pain.

[3] Raftery M.N., Sarma K., Murphy A.W., De la Harpe D., Normand C., McGuire B.E. (2011). Chronic pain in the republic of ireland—Community prevalence, psychosocial profile and predictors of pain-related disability: Results from the prevalence, impact and cost of chronic pain (prime) study, part 1. Pain.

[4] Elliott A., Smith B., Kay P., Smith C., Chambers A. (1999). The epidemiology of chronic pain in the community. Lancet.

[5] Gibson S., Katz B., Corran T., Farrell M., Helme R. (1994). Pain in older adults. Disabil. Rehabil..

[6] Gooberman-Hill R., Heathcote C., Reid C.M., Horwood J., Beswick A.D., Williams S., Ridd M.J. (2011). Professional experience guides opioid prescribing for chronic joint pain in primary care. Fam. Pract..

[7] Fullen B., Hurley D., Power C., Canavan D., O’Keeffe D. (2006). The need for a national strategy for chronic pain management in ireland. Ir. J. Med. Sci..

[8] Green C.R., Wheeler J.R.C., Marchant B., LaPorte F., Guerrero E. (2001). Analysis of the physician variable in pain management. Pain Med..

[9] Kaye A.D., Baluch A., Scott J.T. (2010). Pain management in the elderly population: A review. Ochsner J..

[10] Milton J.C., Hill-Smith I., Jackson S.H. (2008). Prescribing for older people. BMJ.

[11] Hilmer S.N., McLachlan A.J., Le Couteur D.G. (2007). Clinical pharmacology in the geriatric patient. Fundam. Clin. Pharmacol..

[12] OECD Elderly population (indicator). https://data.oecd.org/pop/elderly-population.htm.

[13] Helme R.D. (2001). Chronic pain management in older people. Eur. J. Pain.

[14] Scascighini L., Sprott H. (2008). Chronic nonmalignant pain: A challenge for patients and clinicians. Nat. Clin. Pract. Rheumaol..

[15] Leung L. (2012). From ladder to platform: A new concept for pain management. J. Prim. Health Care.

[16] Benjamin R., Trescot A., Datta S., Buenaventura R., Adlaka R., Sehgal N., Glaser S., Vallejo R. (2008). Opioid complications and side-effects. Pain Phys..

[17] Rosser B.A., McCracken L.M., Velleman S.C., Boichat C., Eccleston C. (2011). Concerns about medication and medication adherence in patients with chronic pain recruited from general practice. PAIN.

[18] McCracken L.M., Velleman S.C., Eccleston C. (2008). Patterns of prescription and concern about opioid analgesics for chronic non-malignant pain in general practice. Prim. Health Care Res. Dev..

[19] Hannes K., Leys M., Vermeire E., Aertgeerts B., Buntinx F., Depoorter A.-M. (2005). Implementing evidence-based medicine in general practice: A focus group based study. BMC Fam. Pract..

[20] Scoggins A., Tiessen J., Ling T., Rabinovich L. (2006). Understanding What shapes Gps’ Prescribing Choices and How might These Be Changed.

[21] McLachlan A.J., Bath S., Naganathan V., Hilmer S.N., Le Couteur D.G., Gibson S.J., Blyth F.M. (2011). Clinical pharmacology of analgesic medicines in older people: Impact of frailty and cognitive impairment. Br. J. Clin. Pharmacol..

[22] Nikolaus T., Zeyfang A. (2004). Pharmacological treatments for persistent non-malignant pain in older persons. Drugs Aging.

[23] Berry P., Chapman R., Covington E., Dahl J., Katz J., Miaskowski C., McLean M. (2001). Pain: Current Understanding of Assessment, Management and Treatments.

[24] Cleeland C.S., Cleeland L.M., Dar R., Rinehardt L.C. (1986). Factors influencing physician management of cancer pain. Cancer.

[25] Upshur C.C., Luckmann R.S., Savageau J.A. (2006). Primary care provider concerns about management of chronic pain in community clinic populations. J. Gen. Intern. Med..

[26] Nolan A., Nolan B. (2004). Ireland’s healthcare system: Some issues and challenges. ESRI Working Paper No. 14.

[27] Barry M.J., Edgman-Levitan S. (2012). Shared decision making—The pinnacle of patient-centered care. N. Engl. J. Med..

[28] International association for the study of pain (2005). Time to modify the who analgesic ladder?. Pain Clin. Updates.

[29] Shaneyfelt T.M. (1999). Are guidelines following guidelines?: The methodological quality of clinical practice guidelines in the peer-reviewed medical literature. J. Am. Med. Assoc..

[30] Cabana M.D., Rand C.S., Powe N.R., Wu A.W., Wilson M.H., Abboud P.-A.C., Rubin H.R. (1999). Why don’t physicians follow clinical practice guidelines? A framework for improvement. J. Am. Med. Assoc..

[31] Shaneyfelt T.M., Centor R.M. (2009). Reassessment of clinical practice guidelines: Go gently into that good night. J. Am. Med. Assoc..

[32] Francis J.J., Johnston M., Robertson C., Glidewell L., Entwistle V., Eccles M.P., Grimshaw J.M. (2009). What is an adequate sample size? Operationalising data saturation for theory-based interview studies. Psychol. Health.

[33] Smith J., Firth J. (2011). Qualitative data analysis: The framework approach. Nurse Res..

[34] Guest G., MacQueen K., Namey E. (2012). Introduction to applied thematic analysis. Applied Thematic Analysis.

[35] Kawulich B. (2004). Data analysis techniques in qualitative research. J. Res. Educ..

[36] Johnson R.B. (1997). Examining the validity structure of qualitative research. Education.

[37] Barbour R.S. (2001). Checklists for improving rigour in qualitative research: A case of the tail wagging the dog?. BMJ Br. Med. J..

[38] Rosemann T., Szecsenyi J. (2004). General practitioners’ attitudes towards research in primary care: Qualitative results of a cross sectional study. BMC Fam. Pract..

[39] Boger E.J., Jones A.K.P. (2005). Paracetamol use in musculoskeletal pain: An audit of use and patient perceptions of paracetamol as an effective analgesic. Musculoskelet. Care.

[40] Magin P., Goode S., Pond D. (2015). Gps, medications and older people: A qualitative study of general practitioners’ approaches to potentially inappropriate medications in older people. Australas. J. Ageing.

[41] Mikhail S.S., Zwar N.A., Vagholkar S., Dennis S.M., Day R.O. (2007). Non-steroidal anti-inflammatory drugs in general practice: A decision-making dilemma. Med. J. Aust..

[42] Taylor R., Lemtouni S., Weiss K., Pergolizzi J.V. (2012). Pain management in the elderly: An fda safe use initiative expert panel’s view on preventable harm associated with nsaid therapy. Curr. Gerontol. Geriatr. Res..

[43] Zenz M., Strumpf M., Tryba M. (1992). Long-term oral opioid therapy in patients with chronic nonmalignant pain. J. Pain Symptom. Manag..

[44] Morgan J.P. (1985). American opiophobia: Customary underutilization of opioid analgesics. Adv. Alcohol. Subst. Abus..

[45] Spitz A., Moore A., Papaleontiou M., Granieri E., Turner B., Reid M. (2011). Primary care providers’ perspective on prescribing opioids to older adults with chronic non-cancer pain: A qualitative study. BMC Geriatr..

[46] Auret K., Schug S.A. (2005). Underutilisation of opioids in elderly patients with chronic pain: Approaches to correcting the problem. Drugs Aging.

[47] Weissman D.E., Joranson D.E., Hopwood M.B. (1991). Wisconsin physicians’ knowledge and attitudes about opioid analgesic regulations. Wis. Med. J..

[48] Hutchinson K., Moreland A.M.E., de C. Williams A.C., Weinman J., Horne R. (2007). Exploring beliefs and practice of opioid prescribing for persistent non-cancer pain by general practitioners. Eur. J. Pain.

[49] Scanlon M.N., Chugh U. (2004). Exploring physicians’ comfort level with opioids for chronic noncancer pain. Pain Res. Manag. J. Can. Pain Soc..

[50] Bhamb B., Brown D., Hariharan J., Anderson J., Balousek S., Fleming M.F. (2006). Survey of select practice behaviors by primary care physicians on the use of opioids for chronic pain. Curr. Med. Res. Opin..

[51] McCracken L.M., Boichat C., Eccleston C. (2012). Training for general practitioners in opioid prescribing for chronic pain based on practice guidelines: A randomized pilot and feasibility trial. J. Pain.

[52] Wolfert M.Z., Gilson A.M., Dahl J.L., Cleary J.F. (2010). Opioid analgesics for pain control: Wisconsin physicians’ knowledge, beliefs, attitudes, and prescribing practices. Pain Med..

[53] Hibble A., Kanka D., Pencheon D., Pooles F. (1998). Guidelines in general practice: The new tower of babel?. BMJ.

[54] Mavrocordatos P., Huygen F., Sichere P., Pergolizzi J. (2015). Benefits of the multidisciplinary team for the patient. Towards a Multidisciplinary Team Approach in Chronic Pain Management.

[55] Thomas E.M., Weiss S.M. (2000). Nonpharmacological interventions with chronic cancer pain in adults. Cancer Control J. Moffitt Cancer Center.

[56] Chaudhari S., Feaver S. (2011). Organization of pain management services. Anaesth. Intensive Care Med..

[57] Lansbury G. (2000). Chronic pain management: A qualitative study of elderly people’s preferred coping strategies and barriers to management. Disabil. Rehabil..

[58] Raftery M.N., Ryan P., Normand C., Murphy A.W., de la Harpe D., McGuire B.E. (2012). The economic cost of chronic noncancer pain in ireland: Results from the prime study, part 2. J. Pain.

[59] Novick G. (2008). Is there a bias against telephone interviews in qualitative research?. Res. Nurs. Health.

[60] Frey J., Oishi S. (1995). How to Conduct Interviews by Telephone and in Person.

[61] Fenig S., Levav I., Kohn R., Yelin N. (1993). Telephone vs face-to-face interviewing in a community psychiatric survey. Am. J. Public Health.

[62] Zenz M., Willweber-Strumpf A. (1993). Opiophobia and cancer pain in europe. Lancet.

[63] Grumbach K., Bodenheimer T. (2004). Can health care teams improve primary care practice?. J. Am. Med. Assoc..

